# Psychotic symptoms in the context of levodopa treatment in Parkinson’s Disease: clinical feature or iatrogenesis?

**DOI:** 10.1192/j.eurpsy.2022.1765

**Published:** 2022-09-01

**Authors:** M.L. Costa, A. Cerame

**Affiliations:** 1 Hospital Universitario Severo Ochoa, Psychiatry, Leganes, Spain; 2 Hospital Universitario José Germain, Hospital De Día, Leganes, Spain

**Keywords:** Parkinson disease, PSYCHOTIC DISORDERS, psychopharmacology, psychostimulants

## Abstract

**Introduction:**

We present the case of a 65-year-old woman who was prescribed levodopa by the Neurology department due to motor symptoms that were not clearly suggestive of a diagnosis of Parkinson’s Disease (PD). Before that, the patient had never showed any hallucinations or delusions. After years of increasing doses of levodopa, the diagnosis of PD was withdrawn. However, during the treatment the patient developed severe psychosis with severe impairment of social functioning. Levodopa is the most effective treatment for motor symptoms in PD. However, it might involve difficulties in dose-control and may present with deletereous complications and adverse effects.

**Objectives:**

To analyse the incidence of pharmacological psychotic symptoms after levodopa treatment.

**Methods:**

A case report is presented alongside a review of the available literature regarding psychotic symptoms in patients treated with levodopa.

**Results:**

Hallucinations and delusions are prevalent symptoms of PD. Nonetheless, they could also be potential side-effects of levodopa treatment. When psychotic symptoms occur, they are commonly attributed to the natural course of the disease. Available evidence does not provide with clear guidelines to distinguish the iatrogenic syndrome from the one caused by the disease itself. Our patient, whose PD diagnosis was dismissed, presented extreme psychotic symptoms which disappeared after the discontinuation of levodopa treatment.

**Conclusions:**

Levodopa is an effective treatment with important risks which must not be overlooked. Adverse effects of the drug could have been minimized by making an accurate differential diagnosis of PD. Individualized benefit-risk balance previous to prescription and a close follow-up should be standardized.

**Disclosure:**

No significant relationships.

